# Sensory Characteristics of Probiotic-Containing Foods: A Multidisciplinary Perspective on Enhancing Acceptability and Consumer Adherence

**DOI:** 10.3390/nu18010032

**Published:** 2025-12-21

**Authors:** Alessandro Tonacci, Francesca Gorini

**Affiliations:** Institute of Clinical Physiology, National Research Council, 56124 Pisa, Italy

**Keywords:** acceptability, consumer behavior, food technology, gut microbiota, immunity, functional foods, probiotics, sensory perception

## Abstract

Globally, the consumption of foods containing probiotics has increased significantly due to their well-recognized health benefits, including the modulation of gut microbiota and immune function. However, despite strong scientific support, daily massive adherence to probiotic food remains limited, mainly because of their suboptimal sensory appeal and the huge variability in consumer expectations. Sensory attributes—flavor, aroma, texture, and appearance—strongly influence liking, purchase, and the habitual consumption necessary for probiotics to exert the physiological effects for which they are consumed. The present narrative review explores the complex, multidimensional interplay between sensory features, consumer perception, and probiotic efficacy. By integrating evidence from nutritional science, microbiology, sensory science, and behavioral psychology, we outline how technological innovation and sensory optimization can improve both product acceptability and adherence. We also discuss how cross-modal perception, the cultural framework, and labeling influence hedonic responses. Finally, we highlight emerging directions, such as sensory-driven strain selection, omics-based flavor profiling, and personalized sensory nutrition, as tools to bridge the gap between scientific efficacy and consumer satisfaction. Improving the sensory design of probiotic foods is pivotal to translate microbiome science into meaningful, sustainable dietary behaviors that support the nutrition–gut–immunity axis.

## 1. Introduction

The scientific and commercial interest towards probiotics has grown significantly in recent years [1,2,3]. Defined as “live microorganisms that, when administered in adequate amounts, confer a health benefit on the host” [4], probiotics are now well integrated into several edible products, ranging from yogurts and fermented beverages to plant- and capsule-based formulations. They exert a fundamental role in maintaining gut homeostasis, immune modulation, and metabolic health [5,6,7,8]; however, in order to be effective for the human body, they need to be regularly consumed, since most of these strains fail to permanently colonize the gut [9]. This fact highlights the importance of their adherence to certain sensory characteristics, since the sensory appeal and the overall palatability of edible compounds appear to be pivotal for nutritional therapy adherence [10].

Another particularly important issue for the large-scale adoption of probiotics is the fragmented related legislation. In fact, for example, the European regulatory framework for probiotics, considering a probiotic restrictedly as a health claim unless supported by a claim under the evaluation by the European Food Security Agency, has failed consumers and industry alike, restricting key information on scientifically backed products. This has impeded the differentiation of higher-quality products from the rest, generating distrust in the category, curbing market growth, and thus holding back investments in the probiotics cradle continent, in turn creating confusion between fermented foods, dietary microbes, and probiotics meeting the IPA Europe and International Scientific Association for Probiotics and Prebiotics (ISAPP) criteria, and representing a further hurdle to their distribution and consumption. On the other hand, the United States regulates probiotics under the Dietary Supplement Health and Education Act (DSHEA), allowing a broader marketing flexibility, and Asia-Pacific regions, including Japan and South Korea, allow functional claims under the Foods for Specified Health Uses (FOSHU) standards. Taken together, such fragmentation limits consumer understanding and hinders innovation and commercialization.

However, while the microbiological and physiological effects of probiotics are well known and have been extensively studied, less attention has been paid to the sensory experience associated with their consumption; this is probably the main feature around which researchers can operate to increase their appeal in the market. In some cases, the presence of micro-organisms in such compounds can affect acidity, viscosity, and the formation of flavory metabolites, in turn possibly affecting sensory quality, thus modulating consumer preferences [11,12,13]. Nevertheless, sensory perception is intrinsically multidimensional, as it is influenced by individual differences (taste and smell sensitivity, microbiota composition, etc.), psychological states, and socio-cultural framework [14,15,16,17]. Therefore, the understanding and optimization of sensory attributes is pivotal for promoting the success of a given product and also for the framework of translational nutrition, ensuring the acceptance of probiotic interventions and adherence to this specific nutritional therapy.

Under this light, the purpose of the present narrative review is to outline the available evidence across different disciplines, including nutritional science, microbiology, food technology, sensory analysis, and consumer psychology, to stress how sensory features can influence the acceptability and the continuous consumption of foods containing probiotics. In the document, we further discuss technological and behavioral strategies to enhance sensory appeal while maintaining microbial viability and health efficacy.

## 2. Search Strategy

We performed a structured narrative search using PubMed, Scopus, and Web of Science. Additional gray literature and regulatory documents were also retrieved from the EFSA, FDA, and FAO/WHO repositories. The search included studies published from 2000 to 2025, using terms such as “gut microbiota”, probiotic function”, “probiotic foods”, “sensory characteristics”, “matrix effects”, “probiotic survival”, and “consumer acceptance”.

## 3. Probiotics, Gut Microbiota, and the Immune Interface

The human gut microbiota, comprising approximately 10 to 100 trillion microorganisms and up to 1000 bacterial species, accounts for nearly 1 kg of the total body weight [18]. It plays a pivotal role in maintaining a host’s health by contributing to immune regulation, nutrient metabolism, and antimicrobial protection, promoting tolerance to beneficial commensals and preventing overgrowth of resident pathogens [19,20,21]. The dominant gut microbial phyla include Firmicutes, Bacteroidetes, Actinobacteria, Proteobacteria, Fusobacteria, and Verrucomicrobia, with Firmicutes and Bacteroidetes together representing about 90% of gut microbiota [22]. The Firmicutes phylum comprises more than 200 genera, of which approximately 95% belong to the genus *Clostridium*, followed by *Lactobacillus*, *Bacillus*, *Enterococcus*, and *Ruminococcus*. Bacteroidetes are primarily represented by *Bacteroides* and *Prevotella* genera [22]. Importantly, the Firmicutes-to-Bacteroidetes ratio is considered a key parameter associated with pathological conditions, including obesity and inflammatory bowel disease [23,24].

Overall, the gut microbiota, predominantly located in the lower intestinal tract—including the small and large intestines—establishes a crucial relationship with the gut-associated immune system [25,26]. This interaction contributes to the development of the largest immunological organ in the human body, containing 70% of all immune cells [27]. The immune system can be broadly classified into non-specific innate immunity and specific adaptive immunity [25]. Innate immunity represents the first line of defense against exogenous agents, providing rapid but non-specific protection. It involves a range of cellular components—including dendritic cells (DCs), monocytes, macrophages, neutrophils, and natural killer (NK) cells—as well as soluble mediators such as cytokines, including interleukins (ILs), interferon gamma (IFN-γ), and tumor necrosis factor alpha (TNF-α) [27] (Table 1). Adaptive immunity, in contrast, is highly specific against foreign antigens and can be further divided into humoral and cell-mediated responses [28]. Humoral immunity is primarily mediated by B cells, which produce immunoglobulins (Igs) targeting extracellular pathogens [27,28]. Cell-mediated immunity involves CD4^+^ T cells—differentiated into distinct T helper subsets based on their cytokine secretion profiles—and CD8^+^ cytotoxic T lymphocytes, which directly eliminate infected or malignant cells [27,28] (Table 1). Notably, the intestinal immune system can distinguish between commensal and pathogenic bacteria—a property known as immunological tolerance—which ensures that immune responses are not mounted against resident microorganisms under physiological conditions [27]. This key function is mediated by pattern recognition receptors such as Toll-like receptors (TLRs) and nucleotide-binding oligomerization domain (NOD)-like receptors [27]. TLRs are expressed on the surface of DCs and macrophages, where they play a crucial role in pathogen recognition and the initiation of innate immune responses [29]. In contrast, NOD-like receptors are specialized in monitoring the intracellular environment for signs of infection and toxic substances [30].

Short-chain fatty acids (SCFAs), the most abundant microbial metabolites in the colonic lumen, are produced by the microbial fermentation of dietary fiber and mainly consist of acetate, propionate, butyrate, and valerate [44]. These metabolites modulate immune responses via multiple mechanisms, including inhibition of histone deacetylase, activation of G-protein-coupled receptor (GPR) signaling, and regulation of acetyl-CoA production [44]. Importantly, once produced in the colon, SCFAs are absorbed into the bloodstream and transported through the circulatory system, allowing them to reach distant organs and tissues, where they exert a variety of beneficial effects [45,46].

A wide array of environmental and host-related factors (e.g., changes in dietary patterns and lifestyle, genetic predisposition, age, infections, medication use) can disrupt the microbial ecosystem beyond its capacity for resistance and resilience, leading to alterations in the composition and function of microbiota, a condition referred to as dysbiosis [47,48]. In addition to a reduction or complete loss of commensal microorganisms and decreased microbial richness, a dysbiotic microbiota is characterized by the abnormal expansion of so-called pathobionts, bacterial species (e.g., bacterial family Enterobacteriaceae) that are typically present at low concentrations under physiological conditions but exhibit pathogenic potential when the gut ecosystem is perturbed [48]. Alterations in the gut microbial community impair immune activation through multiple mechanisms, including modulation of TLR signaling and degradation of secretory IgA, ultimately contributing to a bidirectional feedback loop in which the host immune system and the microbiota continuously influence one another [48]. This dynamic interplay has been implicated in the development of immune-mediated diseases such as multiple sclerosis [49], asthma [50], inflammatory bowel disease [51], autoimmune thyroid diseases [52], celiac disease [53], and rheumatoid arthritis [54], although it remains unclear whether gut dysbiosis is a cause or a consequence of detrimental health conditions [55].

**Figure 1:**
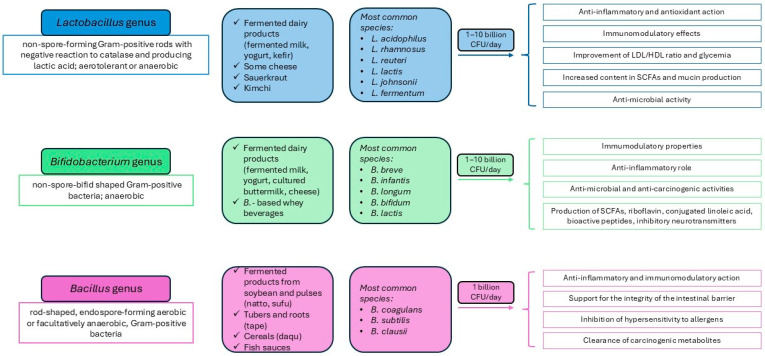
Overview of characteristics, food sources, and key activities of major probiotic groups. Abbreviations: CFU: colony forming unit; HDL: high-density lipoprotein; LDL: low-density lipoprotein; SCFA: short-chain fatty acid.

Nonetheless, the maintenance of a healthy gut microbiota is essential for preventing infections and pathogenic insults, thereby limiting the activation of host immune responses and preserving mucosal homeostasis [56]. According to the 2001 definition by the World Health Organization, probiotics are non-pathogenic living microorganisms that, when administered in adequate amounts, confer health benefits to the host [57]. Currently, up to 35 probiotic species or subspecies have been identified in food products [56]. They are broadly classified into three categories: lactic acid-producing bacteria (e.g., *Lactobacillus*, *Bifidobacterium*, *Enterococcus*); spore-forming *Bacillus* species; and selected strains of *Escherichia coli*, *Streptococcus oralis*, and *Streptococcus salivarius*, together with the yeast species *Saccharomyces boulardii* (reviewed in [27,56,58,59,60,61,62]). Among the numerous microorganisms currently defined as probiotics, *Lactobacillus spp.* (Firmicutes) and *Bifidobacterium spp.* (Actinobacteria) are the most widely used and considered safe in humans [63,64,65,66] (Figure 1). On the other hand, *Lactobacillus* and *Bifidobacterium* are particularly sensitive to the acidic gastric environment and bile salts, and they do not tolerate heat treatment; consequently, their bioavailability may be substantially reduced [67,68]. In contrast, *Bacillus spp.* can withstand extreme temperature and pressure conditions in food processing and remain viable within the digestive tract, persisting for long periods both at room temperature and under refrigeration owing to their ability to form spores [69]. Therefore, *Bacillus spp.* have progressively gained attention and are now available as commercial probiotic supplements, being incorporated into a wide range of food products [67,70,71] (Figure 1).

**Figure 2:**
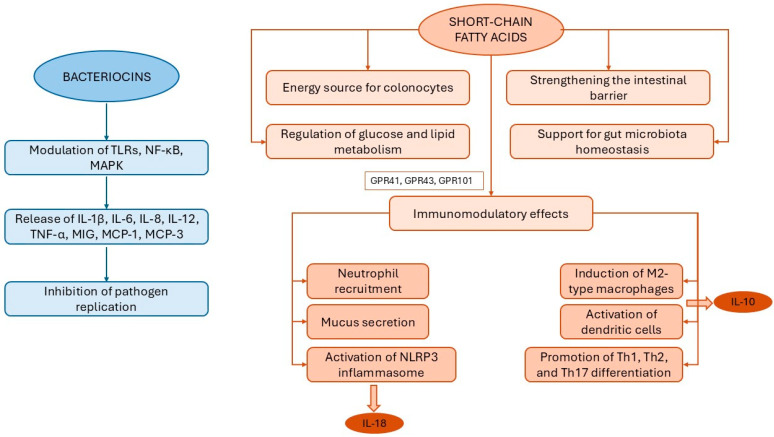
Principal effects of bacteriocins and short-chain fatty acids. Abbreviations: GPR: G-protein-coupled receptor IL: interleukin; MAPK: mitogen-activated protein kinase; NF-κB: nuclear factor kappa-light-chain-enhancer of activated B cells; Th: T helper cell; TLR: Toll-like receptor; TNF-α: tumor necrosis factor alpha.

When administered in sufficient numbers, probiotics have been shown to contribute to the maintenance of microbial balance and to support the immune function [27,56]. Upon colonizing the colon, they produce a great variety of substances, known as postbiotics, and including SCFAs, vitamins, amino acids, enzymes, flavonoids, and exopolysaccharides [46]. Among these, bacteriocins, small cationic molecules (e.g., lactococcin, enterocin, enterolysin, nisin, sublancin, acidocin), typically composed of 30 to 60 amino acids and predominantly secreted by lactic acid bacteria, exert antibiotic and antiviral effects by inhibiting pathogen replication [72]. Although the immunomodulatory properties of bacteriocins are not yet fully elucidated, they are known to stimulate the innate immune response through an increased production of pro- and anti-inflammatory cytokines (e.g., IL-1β, IL-6, IL-8, IL-12, TNF-α) or chemokines (e.g., MIG, MCP-1, MCP-3) [46]. These effects are mediated through the modulation of key signaling pathways, including TLRs, nuclear factor kappa-light-chain-enhancer of activated B cells, and mitogen-activated protein kinase [46] (Figure 2).

In parallel, SCFAs exert a wide range of essential effects on host physiology, including serving as an energy source for colonocytes, reinforcing intestinal barrier integrity, regulating metabolic parameters such as glucose and lipid metabolism, and restoring gut microbiota balance by promoting the growth of beneficial bacteria and suppressing that of harmful microbes [56,73,74,75]. SCFAs also support immune tolerance and play a pivotal role in modulating both innate and adaptive immune response through the activation of GPR41, GPR43, and GPR101 [46,74]. Specifically, they stimulate chemotaxis of neutrophils at sites of inflammation; induce mucin secretion by Goblet cells; and promote the anti-inflammatory M2 phenotype, characterized by the release of IL-10 [46]; SCFAs also activate DCs to enhance IL-10 production by Tregs and IgA secretion by plasma cells; influence the differentiation of naïve CD4^+^ T cells into Th1, Th2, and Th17 cells; and promote the secretion of IL-18 via activation of the NLRP3 inflammasome [46] (Figure 2).

Studies conducted in animal models have revealed promising prospects for the use of probiotics in modulating immune responses and, consequently, in counteracting diseases of diverse origin [27]. In particular, the administration of probiotic mixtures (*Lactobacillus spp.*, *Streptococcus thermophilus* and *Bifidobacterium bifidum*) has demonstrated therapeutic effects in murine models of inflammatory bowel disease, atopic dermatitis, and rheumatoid arthritis. These benefits are associated with an increase in CD4^+^ Foxp3^+^ Tregs and a downregulation of cytokines linked to Th1, Th2, and Th17 responses [76]. Furthermore, the *Lactobacillus casei* BL23 strain has been reported to exert anti-tumor effects through the modulation of IL-2 signaling, which is known to promote proliferation of NK cells and enhance their cytotoxic activity against tumor cells [77,78]. In an HPV-induced cancer model, administration of *Lactibacillus casei* BL23 led to increased IL-2 production, contributing to the suppression of tumor development [77].

Over the years, hundreds of clinical trials have been carried out to evaluate the effectiveness of probiotics in improving immune function and in preventing or managing a broad spectrum of diseases (reviewed in [27]). Volunteers supplemented with 10^9^ live *Bifidobacterium infantis* bacteria per day exhibited increased numbers of Foxp3^+^CD4^+^ T cells in peripheral blood—a hallmark of mucosal immune tolerance—along with enhanced secretion of IL-10, thus supporting the protective role of *Bifidobacterium infantis* in inflammatory diseases [79]. Supplementation with *Lactobacillus rhamnosus* (6 × 10^9^ colony forming unit—CFU/day) or *Bifidobacterium lactis* (9 × 10^9^ CFU/day) during pregnancy resulted in higher IFN-γ levels in cord blood, as well as increased concentrations of IgA and cytokines (i.e., transforming growth factor β1) in breast milk, suggesting that maternal probiotic intake may influence fetal immune development and exert immunomodulatory effects postnatally through bioactive components transferred via breastfeeding [80]. *Lactobacillus rhamnosus*, administered at a dose of 350 mg/day, has also been shown to reduce symptoms of atopic dermatitis in children aged 4–8 months following an 8-week treatment period, with a significant decrease in the mean change in symptom intensity from baseline compared with a placebo [81]. Furthermore, a recent systematic review found that 85% of randomized controlled trials support the use of probiotics (e.g., *Lactobacillus acidophilus*, *Lactobacillus casei*, *Bifidobacterium longum*, or *Lactobacillus rhamnosus*), administered at doses ranging from 10^6^ to 10^11^ CFU/day over periods of 1 to 24 weeks, as effective in managing side effects in adult oncology patients [82]. These effects were observed when probiotics were provided either as single-strain or multi-strain formulations for a minimum of four weeks [82]. Conversely, although *Lactobacillus rhamnosus* GR-1 and *Lactobacillus reuteri* RC-14 are considered among the most promising species for urinary tract infection (UTI) prevention—when administered intravesically or intravaginally at dosages of 10^9^ CFU daily)—a study involving postmenopausal women with spinal cord injury found that oral probiotic therapy using *L. reuteri* RC-14 and *L. rhamnosus* GR-1, or *L. rhamnosus* GG and *Bifidobacterium* BB-12 (each strain at 10^9^ CFU per capsule), did not significantly reduce UTI incidence [83,84].

While probiotics are generally regarded as safe, with an estimated risk of bacteremia of less than 1 case per 1 million individuals, several reports have documented infections associated with probiotic use, raising concerns about their safety [83,85]. Specifically, although *Lactobacillus*-related bacteremia is rare, 180 cases of lactobacillemia have been reported over the past few decades, including 69 cases diagnosed as infective endocarditis [86]. However, patients who develop *Lactobacillus* infections typically have impaired host defenses, severe underlying diseases or organ failure, indwelling venous catheters or a history of surgical intervention, compromised gut barrier integrity, and are often receiving prolonged antibiotic therapy (e.g., β-lactam antibiotics), which is ineffective for lactobacilli [86,87,88,89,90]. Likewise, *Bifidobacterium* has attracted attention due to reports of bacteremia under specific conditions, such as severe heart failure, prematurity, or neonatal intensive care admission [91,92,93,94]. *S. boulardii*-containing probiotics, primarily recommended for the prevention of *Clostridioides difficile*-associated disease, can also be efficiently used for the prevention and treatment of traveler’s diarrhea and for reducing symptoms related to *Helicobacter pylori* treatment [95]. However, in the presence of risk factors, such as admission to intensive care units, central venous lines, or advanced age, their use has been associated with the development of *Saccharomyces cerevisiae* fungemia, which carries an approximately 50% mortality rate [96]. In sum, while in vivo studies support the immunomodulatory effects of probiotics and, consequently, potential beneficial effects on various pathological conditions, clinical data, though promising, highlights the need for further research aimed at optimizing probiotic dosages, treatment duration, and strain-specific efficacy within targeted clinical contexts. In this regard, probiotic-derived compounds, such as bacteriocins and SCFAs, may offer a valid alternative for enhancing immune function without the effects potentially associated with the administration of live microorganisms. Further studies are warranted to validate the biological activity of purified bacteriocins and SCFAs and to assess their clinical impact on host health.

## 4. Sensory Dimensions of Probiotic-Containing Foods

### 4.1. Flavor and Aroma

For human beings, flavor is universally recognized as the most critical determinant of food acceptability, even if the full eating experience is given by the multisensory integration of gustation (taste), olfaction (smell), somatosensory oral perception (texture, chemesthesis), vision, and also audition (e.g., sound of crunchy foods), along with their processing at the brain level [97,98]. During probiotic fermentation, the involvement of beneficial microorganisms like *Lactobacillus*, *Bifidobacterium*, and *Saccharomyces* converts substrates in foods into more bioavailable forms [99,100], in turn producing a plethora of volatile organic compounds that shape the aroma profile [101,102]. Probiotic formulations, for example, often affect the perception of sweetness due to acidification and metabolite interactions, with the sweet notes often altering the sour flavor, but possibly also impacting on the overall salubrity of the compound. Thus, natural sweeteners like stevia or monk fruit, together with proper modulation of fermentation times or co-culturing strategies, can enhance a compound’s palatability without enhancing the amount of sugar content [103]. Therefore, the balance between sensory characteristics and healthiness determines the success of a probiotic, taking into account the reference market, which is not represented by trained panelists or sensory experts but by common citizens, with their own expectancies and personal preferences when it comes to the sensory content of the specific compound proposed.

### 4.2. Texture and Mouthfeel

As stated in the previous paragraph, texture represents another key sensory dimension deeply affecting the perceived quality and satisfaction of a given edible compound, including probiotics [97,98]. In this specific domain, the presence of live cultures can alter several characteristics, including viscosity, and mouth-coating, especially in dairy and plant-based matrices [104,105,106]. For example, yogurts can be enhanced in terms of creaminess and body using probiotic strains with exopolysaccharide (EPS)-producing capacity, improving their overall hedonic ratings [107].

Techniques for microencapsulation, including the use of alginate-, carrageenan-, or lipid-based coatings, can improve textures and survival during processing [108,109]. In non-dairy products, mouthfeel can be modulated using other compounds, including hydrocolloids (pectin, guar gum) or plant proteins (soy, oat, pea), ensuring the maintenance of textural pleasure even after probiotic fortification [110,111,112]. Beyond mouthfeel, such compounds are also known to modulate probiotic viability. For example, polyphenols may provide antioxidant benefits [113], beyond inhibiting the bacterial growth depending on concentration [114], whereas collagen peptides might influence the texture and support the stability of the matrix [115].

Overall, texture perception is not just mechanical, since more complex mechanisms take place, including flavor–texture interactions. For example, even in the presence of low levels of sugar, creaminess enhances the perceived sweetness and aroma intensity, through a combination of texture, fat, and flavor interactions, with a sample cross-modal effect observed in most compounds [116,117].

### 4.3. Appearance and Visual Attributes

In order to build up a fully attractive probiotic product, it is essential to also take into account its visual attributes, which are capable of strongly influencing consumer expectations and the related sensory interpretation. This is particularly true when it comes to probiotic beverages, which present common features like cloudiness, sedimentation, or effervescence that can be interpreted by the consumer as signals of authenticity or spoilage, depending on the cultural framework of the individuals. Also, color stability represents a major issue, since pH-induced pigment degradation, especially present in fruit-based probiotic drinks, can alter consumer perception and therefore the success of the beverage overall [118,119]. This is therefore modified using natural pigments, like anthocyanins or carotenoids, which in turn may also enhance functional features of the drink [120]. Lastly, consumer trust can be also be maintained by properly leveraging innovative packaging solutions and clear labeling, possibly indicating the occurrence of degradation in the products [121,122].

In summary, strategies for sensory optimization related to the different probiotic food categories are outlined in Table 2.

## 5. Technological Strategies for Sensory Optimization

Sensory optimization with respect to probiotic products can be attained through three different yet integrated strategies, including the selection of strains and metabolite profiling, the matrix design and microencapsulation approaches, and the synergy between sweeteners, flavors, and prebiotics.

### 5.1. Strain Selection and Metabolite Profiling

The choice around the probiotic strain is a key milestone in the framework of probiotic success and consumption, as it can influence both metabolic output and the sensory fingerprint. For example, different strains produce completely different tastes and odorous compounds. In this regard, the selection of strains with the desired metabolic profile, or co-culturing complementary species offers an innovative pathway to align the microbiological efficacy of a given compound with sensory harmony and appeal.

Recently, applications of metabolomics, volatilomics, and “omics” in general have enabled an accurate mapping of relevant metabolites to specific edible compounds [139]; this is also happening with probiotics [140,141,142]. The recently established concept of “flavoromics”, already applied in other nutritional scenarios [143,144,145], can lead researchers to develop and select probiotics in a sensory-guided manner, with strains being screened for both health efficacy and overall sensory quality, with a significant impact on consumer preferences and choices [146].

### 5.2. Matrix Design and Microencapsulation

The food matrix can have a two-fold usefulness: it can serve as a carrier and a sensory framework. In general, different food matrices have a large effect on probiotic survival. Notably, dairy products typically provide enhanced buffering capacity during exposure to gastric acidity [126,147,148]. On the other hand, plant-based matrices, including soy, oat, or coconut, display largely variable protection due to differing protein structures, fat levels, and the overall carbohydrate composition [130,149]. Examples for this may include the improved survival of *Lactobacillus rhamnosus* in soy-based yogurts [150,151], whereas *Bifidobacterium longum* appears to be quite stable in dairy products [152], but the production conditions and processing techniques have important effects.

Nevertheless, to date, dairy matrices represent the most common example of food matrices in the probiotics universe, although growing demand for plant-based probiotics has introduced new challenges in the sensory framework applied to probiotics, as they introduce different off-flavors, like beany ones, and a reduction in creaminess [153,154,155]. In that, the fermentation of plant substrates, like oat, soy, or coconut, has the ability to improve both microbial viability and sensory acceptance of the end-products through the formation of palatable flavor compounds.

In general terms, microencapsulation enhances probiotic survival under gastric stress [156], but is also capable of modulating flavor release and masking undesired off-flavors, like bitterness or acidity [157]. Microencapsulation techniques are manifold and include, among others, extrusion, emulsion, fluidized bed, freeze-drying, spray-freezing, spray chilling, electrospraying, and microfluidic. In order to choose the optimal microencapsulation technique, it is necessary to perform a thorough assessment of the probiotics to be encapsulated: the intended application, particularly important when in contact with impactful gastric stressors [158]; product requirements; and a cost–benefit analysis, without affecting the health status of the cells [159,160,161]. Importantly, the chosen technique must not be harmful to the cells.

### 5.3. Sweeteners, Flavors, and Prebiotic Synergy

The overall balancing of sweet taste is key to compensate for the acidic nature of most fermented probiotic products [162,163]. In that, natural sweeteners and fruit components are known to improve palatability without compromising the healthiness of the product profile [164]. The inclusion of prebiotics, such as inulin and galacto-oligosaccharides, can boost the mouthfeel, sweetness, and the overall probiotic survival, making it a very promising trend in the field [165,166,167]. Nevertheless, the inclusion of some prebiotic fibers, metabolized by probiotic strains during storage, can potentially lead to acidification, gas production, and the development of off-flavors, depending on the strain, incubation temperature, and water activity of the product: all factors to be taken into account during formulation. As such, the optimization of flavor through bio-flavoring, with micro-organisms that are engineered or purposely selected depending on their volatile production, represents another frontier, with an eye to consumer perception and overall regulatory aspects [168,169].

### 5.4. Shelf Life and Economic Considerations

The shelf life of probiotic foods varies a lot, usually ranging from a few months to two years, in the case of dry products, with lower shelf lives in the case of liquid or refrigerated foods, with more unstable strains. This duration largely depends on the product’s ingredients, packaging, and storage conditions. Together with this variation, costs are also known to vary significantly, with some stable products usually being less expensive, and fermented or specialty products being more costly; cost is also based upon the number of live organisms contained, their potency, and specialized formulations. Few studies have exactly reported the shelf lives of such products; nevertheless, yogurt is often investigated, with 21 days usually employed as the higher limit for its safe and useful storage [170,171].

## 6. Consumer Perception and Behavioral Determinants

### 6.1. Cultural and Contextual Factors

Probiotic food consumption varies globally. For example, between 20 and 40 kg of fermented dairy products is consumed per person each year in Western countries, of which roughly 40% is yogurt [172]. At the global level, among the most commonly used probiotic products are yogurt, kefir, sauerkraut, kimchi, miso, kombucha, pickles, buttermilk, and natto [173], whose sensory attributes, such as mild acidity, creaminess, sweetness balance, and familiar flavor notes are major determinants of adherence and repeated consumption.

Cultural background is one of the main determinants for the success or non-success of a given edible compound. In fact, it deeply shapes sensory expectations in consumers and individuals in general. For example, Mediterranean populations usually associate mild sourness and fermentation notes with freshness and tradition [174], whereas in other parts of the globe, including Northern Europe and North America, the same characteristics are normally associated with spoilage [175]. Therefore, prior knowledge of such cultural differences and beliefs would enable manufacturers to properly tailor their products to the specific market scenario addressed, thus attaining optimized success probabilities. In this regard, cross-cultural sensory mapping and ethnographic studies have revealed that consumer education—including emphasizing probiotic health benefits—is capable of shifting consumer perception from aversion to acceptance [176]. Therefore, communication and sensory framing can be as important as formulation itself.

### 6.2. Expectation, Familiarity, and Trust

Sensory evaluation can be strongly affected by expectations. In fact, when dealing with a product that is labeled as “healthy”, especially when the factory or the product itself is “trusted”, consumers might also accept some sensory features otherwise not desired, including sour or bitter notes, if counterbalanced by the perceived efficacy of the compound for promoting health. In such cases, when consumers believe a product is health-promoting, their brain’s reward responses to its sensory attributes increase [177]. At the same time, familiar products are normally perceived as more pleasant, since, as demonstrated, repeated exposure to a given compound enhances its pleasantness in an exposure adaptation process [178,179]. Therefore, expectations, trust, and familiarity are all dimensions to be considered when producing and commercializing a probiotic or other edible compound.

### 6.3. Cross-Modal Perception and Sensory Integration

Modern sensory science recognizes the cross-modal nature of perception. As such, taste, smell, texture, color, and even sound interact to create the overall sensory experience [180,181]. For example, creamy textures are known to amplify the perception of sweetness [116,117], bright colors are associated with fruity characteristics [182], and also packaging sound can modulate the feeling around the freshness of a product [183]. Relying on such principles could enable sensory compensation strategies, for example, reducing sugar or fat contents, without compromising pleasure, thus optimizing the success of a product and, eventually, the adherence to a nutritional therapeutic plan, overall, even across age groups [184].

## 7. Conclusions and Future Directions

The seamless integration of sensory science with research into the microbiome represents a frontier in the field of functional food design. In that, future efforts should combine the application of omics technologies, including genomics, proteomics, and metabolomics, with sensory and emotional analysis to identify microbial species and associated metabolites in charge of specific sensory signatures, to ultimately enable the predictive modeling of how the formulation of fermentation variables could influence both the flavor and the related health outcomes.

Personalized nutrition, based on sensory cues, such as tailoring probiotic formulations to personal sensory profiles and microbiota composition, is rapidly emerging as a new, transformative concept. Digital tools, mainly those exploiting AI methods and principles, can enable a quick acceleration in the development of consumer-centric products. Trends in sustainability, including low-carbon production chains, and plant-based alternatives (oat, almond, rice, legumes), will also contribute to redefining probiotic sensory challenges and require novel fermentation strategies for the optimization of flavor and texture while supporting the viability and shelf life of probiotics. The early integration of sensory expertise into the formulation will be a cornerstone to ensure overall consumer satisfaction and ecological responsibility and trust.

From a public health perspective, improvements in the sensory appeal of probiotic foods can support long-term adherence to therapies based on functional foods, translating microbiome science into trustworthy, tangible benefits for citizens when it comes to immunity and metabolic resilience. Therefore, sensory optimization should be regarded as a nutritional and immunological enabler beyond its marketing value.

In conclusion, sensory features are pivotal to the success of probiotic-containing foods. Flavor, texture, and appearance, together with psychological, cultural, and contextual factors, can determine consumer acceptability and habitual intake. Since their efficacy largely depends on their regular consumption, sensory optimization is key to the success of health outcomes. The overall process largely depends on an integrated, multidisciplinary approach, linking microbial science, sensory design, food technology, neuroscience, biomedical engineering, and consumer psychology to develop probiotic products that are both biologically effective and sensorially enjoyable. Advances in strain selection, food engineering, and cross-modal design are promising for achieving this integration. In parallel, the harmonization and opening of national and transnational legislations is urgently needed to allow manufacturers to keep investing and progressing in these categories, without risking creating a global divide between markets, with negative consequences not only on the economic and financial side, but also when it comes to health promotion for citizens.

## Figures and Tables

**Table 1:** Main characteristics of the constituents of the intestinal immune system.

Cell Type	Branch of Immunity	Functions	Molecules Released	References
Dendritic cells	Innate	Immune response against pathogens; maintenance of immune homeostasis; antigen-presenting cells	AMPs, cytokines, chemokines	[31,32,33]
Macrophages	Innate	Antigen-presenting cells; pathogen elimination (M1 phenotype); tissue repair (M2 phenotype)	AMPs; IL-1β, IL-6, IL-12α, IL-23, TNF-α (M1 phenotype); IL-10 (M2 phenotype)	[31,34]
Innate lymphoid cells	Innate	Antimicrobial defense; tissue regeneration	IFN-γ (ILC-1); IL-5, IL-9, IL-13 (ILC-2); IL17, IL-23 (ILC-3)	[35,36,37]
Natural killer cells	Innate	Antimicrobial defense	IFN-γ	[38]
Th1 cells	Adaptive	Immunity to intracellular pathogens	IFN-γ, TNF-α, IL-2	[31,33,39,40]
Th2 cells	Adaptive	Elimination of extracellular parasites	IL-4, IL-5, IL-13	[34,39,40]
Th17 cells	Adaptive	Protection against bacteria and fungi	lL-17, IL-22, IL-23, TNF-α	[31,40,41]
Treg cells	Adaptive	Promotion of immune tolerance; suppression of Th17 cell-mediated responses	IL-10, TGF-β	[31,39,40]
Cytotoxic T cells	Adaptive	Elimination of virally infected cells and cancerous cells	IFN-γ, TNF-α, perforin, granzymes	[28]
Plasma cells	Adaptive	Defense against pathogenic bacteria, enhancement of immune tolerance, complement activation, cellular cytotoxicity	IgA, IgG	[42,43]

Abbreviations: AMP: antimicrobial peptide; IFN-γ: interferon gamma; Ig: immunoglobulin; IL: interleukin; ILC: innate lymphoid cell; TGF-β: transforming growth factor beta; Th: T helper; TNF-α: tumor necrosis factor alpha; Treg: T regulatory.

**Table 2:** Overview of Probiotic Food Categories and Sensory Optimization Strategies.

Probiotic Food Category	Key Sensory Attributes	Common Optimization Strategies	Bacteria Included	References
Fermented dairy (yogurt, kefir)	Creaminess, acidity, aroma balance	Starter culture selection; fat/protein modulation; controlled fermentation	*Lactobacillus*, *Streptococcus thermophilus*, *Bifidobacterium*	[123,124,125,126]
Plant-based fermented beverages (soy, oat, coconut)	Viscosity, vegetal notes, sweetness	Flavor masking; enzymatic treatments; stabilizers; strain selection	*Lactobacillus rhamnosus*, *L. casei*, *L. plantarum*	[127,128,129,130]
Fermented vegetables (kimchi, sauerkraut)	Crunchiness, acidity, aroma complexity	Salt concentration control; co-fermentation species; temperature modulation	*Lactiplantibacillus plantarum*	[131,132]
Probiotic juices	Freshness; sweetness–acidity balance	Microencapsulation; pH control; strain selection	*Lactobacillus*, *Bifidobacterium*	[133,134]
Probiotic snacks (bars, baked goods)	Texture stability, flavor integration	Post-bake inoculation; protective matrices; water activity control	Bacterial strains resistant to low water activity	[135]
Functional dairy alternatives (plant yogurts)	Creaminess, mouthfeel, flavor uniformity	Hydrocolloids; fermentation optimization; probiotic–fiber interactions	*Lactobacillus rhamnosus*, *L. casei*, *L. plantarum*	[136,137,138]

## Data Availability

No new data were created or analyzed in this study.

## Abbreviations

The following abbreviations are used in this manuscript:

DC	Dendritic cell
DSHEA	Dietary Supplement Health and Education Act
EFSA	European Food Safety Authority
EPS	Exopolysaccharide
FOSHU	Foods for Specified Health Uses
GPR	G-protein-coupled receptor
IFN-γ	Interferon gamma
Ig	Immunoglobulin
IL	Interleukin
ISAPP	International Scientific Association for Probiotics and Prebiotics
NK	Natural killer
NOD	Nucleotide-binding oligomerization domain
SCFA	Short-chain fatty acid
TLR	Toll-like receptor
TNF-α	Tumor necrosis factor alpha

## References

[1] Stanton C., Gardiner G., Meehan H., Collins K., Fitzgerald G., Lynch P.B., Ross R.P. (2001). Market potential for probiotics. Am. J. Clin. Nutr..

[2] Saxelin M. (2008). Probiotic formulations and applications, the current probiotics market, and changes in the marketplace: A European perspective. Clin. Infect. Dis..

[3] Liang D., Wu F., Zhou D., Tan B., Chen T. (2024). Commercial probiotic products in public health: Current status and potential limitations. Crit. Rev. Food Sci. Nutr..

[4] Hill C., Guarner F., Reid G., Gibson G.R., Merenstein D.J., Pot B., Morelli L., Canani R.B., Flint H.J., Salminen S. (2014). Expert consensus document. The International Scientific Association for Probiotics and Prebiotics consensus statement on the scope and appropriate use of the term probiotic. Nat. Rev. Gastroenterol. Hepatol..

[5] Kaur H., Ali S.A. (2022). Probiotics and gut microbiota: Mechanistic insights into gut immune homeostasis through TLR pathway regulation. Food Funct..

[6] Prosperi M., Santocchi E., Guiducci L., Frinzi J., Morales M.A., Tancredi R., Calderoni S. (2022). Interventions on microbiota: Where do we stand on a gut–brain link in autism? A systematic review. Nutrients.

[7] Yeşilyurt N., Yılmaz B., Ağagündüz D., Capasso R. (2021). Involvement of probiotics and postbiotics in the immune system modulation. Biologics.

[8] Bock P.M., Martins A.F., Schaan B.D. (2024). Understanding how pre- and probiotics affect the gut microbiome and metabolic health. Am. J. Physiol. Endocrinol. Metab..

[9] Zmora N., Zilberman-Schapira G., Suez J., Mor U., Dori-Bachash M., Bashiardes S., Elinav E. (2018). Personalized gut mucosal colonization resistance to empiric probiotics is associated with unique host and microbiome features. Cell.

[10] Lester S., Kleijn M., Cornacchia L., Hewson L., Taylor M.A., Fisk I. (2022). Factors affecting adherence, intake, and perceived palatability of oral nutritional supplements: A literature review. J. Nutr. Health Aging.

[11] Karimi R., Sohrabvandi S., Mortazavian A.M. (2012). Sensory characteristics of probiotic cheese. Compr. Rev. Food Sci. Food Saf..

[12] Conti-Silva A.C., de Souza-Borges P.K. (2019). Sensory characteristics, brand and probiotic claim on the overall liking of commercial probiotic fermented milks: Which one is more relevant?. Food Res. Int..

[13] Ranadheera C.S., Evans C.A., Baines S.K., Balthazar C.F., Cruz A.G., Esmerino E.A., Vasiljevic T. (2019). Probiotics in goat milk products: Delivery capacity and ability to improve sensory attributes. Compr. Rev. Food Sci. Food Saf..

[14] Thangaleela S., Sivamaruthi B.S., Kesika P., Bharathi M., Chaiyasut C. (2022). Nasal microbiota, olfactory health, neurological disorders and aging—A review. Microorganisms.

[15] Bochicchio V., Winsler A. (2020). The psychology of olfaction: A theoretical framework with research and clinical implications. Psychol. Rev..

[16] Caballero R., Paradis C. (2015). Making sense of sensory perceptions across languages and cultures. Funct. Lang..

[17] Kurczewska E., Ferensztajn-Rochowiak E., Rybakowski J., Rybakowski F. (2024). Sensory processing sensitivity as a trait of temperament—Evolutionary, socio-cultural, biological context and relation to mental disorders. Psychiatr. Pol..

[18] Geng J., Ni Q., Sun W., Li L., Feng X. (2022). The links between gut microbiota and obesity and obesity related diseases. Biomed. Pharmacother..

[19] Jandhyala S.M., Talukdar R., Subramanyam C., Vuyyuru H., Sasikala M., Nageshwar Reddy D. (2015). Role of the normal gut microbiota. World J. Gastroenterol..

[20] Adak A., Khan M.R. (2019). An insight into gut microbiota and its functionalities. Cell. Mol. Life Sci..

[21] Kamada N., Chen G.Y., Inohara N., Núñez G. (2013). Control of pathogens and pathobionts by the gut microbiota. Nat. Immunol..

[22] Rinninella E., Raoul P., Cintoni M., Franceschi F., Miggiano G.A.D., Gasbarrini A., Mele M.C. (2019). What is the Healthy Gut Microbiota Composition? A Changing Ecosystem across Age, Environment, Diet, and Diseases. Microorganisms.

[23] Mariat D., Firmesse O., Levenez F., Guimarăes V., Sokol H., Doré J., Corthier G., Furet J.P. (2009). The Firmicutes/Bacteroidetes ratio of the human microbiota changes with age. BMC Microbiol..

[24] Magne F., Gotteland M., Gauthier L., Zazueta A., Pesoa S., Navarrete P., Balamurugan R. (2020). The Firmicutes/Bacteroidetes Ratio: A Relevant Marker of Gut Dysbiosis in Obese Patients?. Nutrients.

[25] Ashaolu T.J. (2020). Immune boosting functional foods and their mechanisms: A critical evaluation of probiotics and prebiotics. Biomed. Pharmacother..

[26] Zhou P., Chen C., Patil S., Dong S. (2024). Unveiling the therapeutic symphony of probiotics, prebiotics, and postbiotics in gut-immune harmony. Front. Nutr..

[27] Mazziotta C., Tognon M., Martini F., Torreggiani E., Rotondo J.C. (2023). Probiotics Mechanism of Action on Immune Cells and Beneficial Effects on Human Health. Cells.

[28] Andersen M.H., Schrama D., Thor Straten P., Becker J.C. (2006). Cytotoxic T cells. J. Investig. Dermatol..

[29] Wang W., Mu S., Yan D., Qin H., Zheng Z. (2025). Comprehending toll-like receptors: Pivotal element in the pathogenesis of sepsis and its complications. Front. Immunol..

[30] Zhong Y., Kinio A., Saleh M. (2013). Functions of NOD-Like Receptors in Human Diseases. Front. Immunol..

[31] Lu Q., Yang M.F., Liang Y.J., Xu J., Xu H.M., Nie Y.Q., Wang L.S., Yao J., Li D.F. (2022). Immunology of Inflammatory Bowel Disease: Molecular Mechanisms and Therapeutics. J. Inflamm. Res..

[32] Zanna M.Y., Yasmin A.R., Omar A.R., Arshad S.S., Mariatulqabtiah A.R., Nur-Fazila S.H., Mahiza M.I.N. (2021). Review of Dendritic Cells, Their Role in Clinical Immunology, and Distribution in Various Animal Species. Int. J. Mol. Sci..

[33] Sun D., Li C., Chen S., Zhang X. (2022). Emerging Role of Dendritic Cell Intervention in the Treatment of Inflammatory Bowel Disease. BioMed Res. Int..

[34] Zhang K., Guo J., Yan W., Xu L. (2023). Macrophage polarization in inflammatory bowel disease. Cell Commun. Signal..

[35] Nagasawa M., Spits H., Ros X.R. (2018). Innate Lymphoid Cells (ILCs): Cytokine Hubs Regulating Immunity and Tissue Homeostasis. Cold Spring Harb. Perspect. Biol..

[36] Saez A., Gomez-Bris R., Herrero-Fernandez B., Mingorance C., Rius C., Gonzalez-Granado J.M. (2021). Innate Lymphoid Cells in Intestinal Homeostasis and Inflammatory Bowel Disease. Int. J. Mol. Sci..

[37] Panda S.K., Colonna M. (2019). Innate Lymphoid Cells in Mucosal Immunity. Front. Immunol..

[38] Poggi A., Benelli R., Venè R., Costa D., Ferrari N., Tosetti F., Zocchi M.R. (2019). Human Gut-Associated Natural Killer Cells in Health and Disease. Front. Immunol..

[39] Guan Q. (2019). A Comprehensive Review and Update on the Pathogenesis of Inflammatory Bowel Disease. J. Immunol. Res..

[40] Raphael I., Nalawade S., Eagar T.N., Forsthuber T.G. (2015). T cell subsets and their signature cytokines in autoimmune and inflammatory diseases. Cytokine.

[41] Jiang P., Zheng C., Xiang Y., Malik S., Su D., Xu G., Zhang M. (2023). The involvement of TH17 cells in the pathogenesis of IBD. Cytokine Growth Factor Rev..

[42] Fleming A., Castro-Dopico T., Clatworthy M.R. (2022). B cell class switching in intestinal immunity in health and disease. Scand. J. Immunol..

[43] Castro-Dopico T., Colombel J.F., Mehandru S. (2020). Targeting B cells for inflammatory bowel disease treatment: Back to the future. Curr. Opin. Pharmacol..

[44] Liu X.F., Shao J.H., Liao Y.T., Wang L.N., Jia Y., Dong P.J., Liu Z.Z., He D.D., Li C., Zhang X. (2023). Regulation of short-chain fatty acids in the immune system. Front. Immunol..

[45] Hanus M., Parada-Venegas D., Landskron G., Wielandt A.M., Hurtado C., Alvarez K., Hermoso M.A., López-Köstner F., De la Fuente M. (2021). Immune System, Microbiota, and Microbial Metabolites: The Unresolved Triad in Colorectal Cancer Microenvironment. Front. Immunol..

[46] Thoda C., Touraki M. (2023). Immunomodulatory Properties of Probiotics and Their Derived Bioactive Compounds. Appl. Sci..

[47] Wen L., Duffy A. (2017). Factors Influencing the Gut Microbiota, Inflammation, and Type 2 Diabetes. J. Nutr..

[48] Levy M., Kolodziejczyk A.A., Thaiss C.A., Elinav E. (2017). Dysbiosis and the immune system. Nat. Rev. Immunol..

[49] Correale J., Hohlfeld R., Baranzini S.E. (2022). The role of the gut microbiota in multiple sclerosis. Nat. Rev. Neurol..

[50] Logoń K., Świrkosz G., Nowak M., Wrześniewska M., Szczygieł A., Gomułka K. (2023). The Role of the Microbiome in the Pathogenesis and Treatment of Asthma. Biomedicines.

[51] Gorini F., Tonacci A. (2025). Selenium: A Key Element in Inflammatory Bowel Disease. Antioxidants.

[52] Gorini F., Tonacci A. (2024). Vitamin D: An Essential Nutrient in the Dual Relationship between Autoimmune Thyroid Diseases and Celiac Disease—A Comprehensive Review. Nutrients.

[53] Rossi R.E., Dispinzieri G., Elvevi A., Massironi S. (2023). Interaction between Gut Microbiota and Celiac Disease: From Pathogenesis to Treatment. Cells.

[54] Ermencheva P., Kotov G., Shumnalieva R., Velikova T., Monov S. (2024). Exploring the Role of the Microbiome in Rheumatoid Arthritis—A Critical Review. Microorganisms.

[55] McBurney M.I., Davis C., Fraser C.M., Schneeman B.O., Huttenhower C., Verbeke K., Walter J., Latulippe M.E. (2019). Establishing What Constitutes a Healthy Human Gut Microbiome: State of the Science, Regulatory Considerations, and Future Directions. J. Nutr..

[56] Liu Y., Wang J., Wu C. (2022). Modulation of Gut Microbiota and Immune System by Probiotics, Prebiotics, and Postbiotics. Front. Nutr..

[57] Mack D.R. (2005). Probiotics—Mixed messages. Can. Fam. Physician.

[58] Rastogi S., Singh A. (2022). Gut microbiome and human health: Exploring how the probiotic genus *Lactobacillus* modulate immune responses. Front. Pharmacol..

[59] Widyastuti Y., Febrisiantosa A., Tidona F. (2021). Health-Promoting Properties of Lactobacilli in Fermented Dairy Products. Front. Microbiol..

[60] Sibanda T., Marole T.A., Thomashoff U.L., Thantsha M.S., Buys E.M. (2024). *Bifidobacterium* species viability in dairy-based probiotic foods: Challenges and innovative approaches for accurate viability determination and monitoring of probiotic functionality. Front. Microbiol..

[61] Jena R., Choudhury P.K. (2025). Bifidobacteria in Fermented Dairy Foods: A Health Beneficial Outlook. Probiotics Antimicrob. Proteins.

[62] Turnbull P.C.B., Baron S. (1996). Bacillus. Medical Microbiology.

[63] Matera M. (2024). *Bifidobacteria*, *Lactobacilli*… when, how and why to use them. Glob. Pediatr..

[64] Cheng H., Ma Y., Liu X., Tian C., Zhong X., Zhao L. (2022). A Systematic Review and Meta-Analysis: *Lactobacillus acidophilus* for Treating Acute Gastroenteritis in Children. Nutrients.

[65] Pedret A., Valls R.M., Calderón-Pérez L., Llauradó E., Companys J., Pla-Pagà L., Moragas A., Martín-Luján F., Ortega Y., Giralt M. (2019). Effects of daily consumption of the probiotic *Bifidobacterium animalis* subsp. lactis CECT 8145 on anthropometric adiposity biomarkers in abdominally obese subjects: A randomized controlled trial. Int. J. Obes..

[66] Hoa V.B., Park S.H., Ha D.H., Son J.H., Lee K.H., Park W.S., Yoo J.Y., Bae I.S., Kim H.W., Kang H.B. (2024). Daily Supplementation with *Bifidobacterium longum KACC91563* Alleviates Allergic Contact Dermatitis in an Animal Model. Foods.

[67] Payne J., Bellmer D., Jadeja R., Muriana P. (2024). The Potential of *Bacillus* Species as Probiotics in the Food Industry: A Review. Foods.

[68] Konuray G., Erginkaya Z. (2018). Potential Use of *Bacillus coagulans* in the Food Industry. Foods.

[69] Dastjerdi Z.K., Ghazi S. (2025). Microencapsulation of *Bacillus Mojavensis* as potent probiotic strain isolated from soil of Iran and determining characterization. Microbe.

[70] Li Z., Zheng M., Zheng J., Gänzle M.G. (2023). *Bacillus* species in food fermentations: An underappreciated group of organisms for safe use in food fermentations. Curr. Opin. Food Sci..

[71] Dang H.T., Tran D.M., Phung T.T.B., Bui A.T.P., Vu Y.H., Luong M.T., Nguyen H.M., Trinh H.T., Nguyen T.T., Nguyen A.H. (2024). Promising clinical and immunological efficacy of *Bacillus clausii* spore probiotics for supportive treatment of persistent diarrhea in children. Sci. Rep..

[72] Youssef M., Ahmed H.Y., Zongo A., Korin A., Zhan F., Hady E., Umair M., Shahid Riaz Rajoka M., Xiong Y., Li B. (2021). Probiotic Supplements: Their Strategies in the Therapeutic and Prophylactic of Human Life-Threatening Diseases. Int. J. Mol. Sci..

[73] Aho V.T.E., Houser M.C., Pereira P.A.B., Chang J., Rudi K., Paulin L., Hertzberg V., Auvinen P., Tansey M.G., Scheperjans F. (2021). Relationships of gut microbiota, short-chain fatty acids, inflammation, and the gut barrier in Parkinson’s disease. Mol. Neurodegener..

[74] Kim C.H. (2023). Complex regulatory effects of gut microbial short-chain fatty acids on immune tolerance and autoimmunity. Cell. Mol. Immunol..

[75] Mansuy-Aubert V., Ravussin Y. (2023). Short chain fatty acids: The messengers from down below. Front. Neurosci..

[76] Kwon H.K., Lee C.G., So J.S., Chae C.S., Hwang J.S., Sahoo A., Nam J.H., Rhee J.H., Hwang K.C., Im S.H. (2010). Generation of regulatory dendritic cells and CD4^+^Foxp3^+^ T cells by probiotics administration suppresses immune disorders. Proc. Natl. Acad. Sci. USA.

[77] Jacouton E., Michel M.L., Torres-Maravilla E., Chain F., Langella P., Bermúdez-Humarán L.G. (2019). Elucidating the Immune-Related Mechanisms by Which Probiotic Strain *Lactobacillus casei* BL23 Displays Anti-tumoral Properties. Front. Microbiol..

[78] Kim N., Yi E., Lee E., Park H.J., Kim H.S. (2024). Interleukin-2 is required for NKp30-dependent NK cell cytotoxicity by preferentially regulating NKp30 expression. Front. Immunol..

[79] Konieczna P., Groeger D., Ziegler M., Frei R., Ferstl R., Shanahan F., Quigley E.M., Kiely B., Akdis C.A., O’Mahony L. (2012). *Bifidobacterium infantis* 35624 administration induces Foxp3 T regulatory cells in human peripheral blood: Potential role for myeloid and plasmacytoid dendritic cells. Gut.

[80] Prescott S.L., Wickens K., Westcott L., Jung W., Currie H., Black P.N., Stanley T.V., Mitchell E.A., Fitzharris P., Siebers R. (2008). Supplementation with *Lactobacillus rhamnosus* or *Bifidobacterium lactis* probiotics in pregnancy increases cord blood interferon-γ and breast milk transforming growth factor-β and immunoglobulin A detection. Clin. Exp. Allergy.

[81] Wu Y.J., Wu W.F., Hung C.W., Ku M.S., Liao P.F., Sun H.L., Lu K.H., Sheu J.N., Lue K.H. (2017). Evaluation of efficacy and safety of *Lactobacillus rhamnosus* in children aged 4–48 months with atopic dermatitis: An 8-week, double-blind, randomized, placebo-controlled study. J. Microbiol. Immunol. Infect..

[82] Rodriguez-Arrastia M., Martinez-Ortigosa A., Rueda-Ruzafa L., Folch Ayora A., Ropero-Padilla C. (2021). Probiotic supplements on oncology patients’ treatment-related side effects: A systematic review of randomized controlled trials. Int. J. Environ. Res. Public Health.

[83] Falagas M.E., Betsi G.I., Tokas T., Athanasiou S. (2006). Probiotics for prevention of recurrent urinary tract infections in women: A review of the evidence from microbiological and clinical studies. Drugs.

[84] Toh S.L., Lee B.B., Ryan S., Simpson J.M., Clezy K., Bossa L., Rice S.A., Marial O., Weber G.H., Kaur J. (2019). Probiotics (LGG-BB12 or RC14-GR1) versus placebo as prophylaxis for urinary tract infection in persons with spinal cord injury (ProSCIUTTU): A randomised controlled trial. Spinal Cord.

[85] Mackay A.D., Taylor M.B., Kibbler C.C., Hamilton-Miller J.M. (1999). *Lactobacillus* endocarditis caused by a probiotic organism. Clin. Microbiol. Infect..

[86] Borriello S.P., Hammes W.P., Holzapfel W., Marteau P., Schrezenmeir J., Vaara M., Valtonen V. (2003). Safety of probiotics that contain lactobacilli or bifidobacteria. Clin. Infect. Dis..

[87] Cornacchiari M., Mudoni A., Liccardo A., Visciano B., Rizzo M.A., Cuoccio P., Di Toma L.F. (2024). *Lactobacillemia*: A Rare Entity in Immunocompromised Patients. Description of a Clinical Case and Literature Review. G. Ital. Nefrol..

[88] Vahabnezhad E., Mochon A.B., Wozniak L.J., Ziring D.A. (2013). *Lactobacillus bacteremia* associated with probiotic use in a pediatric patient with ulcerative colitis. J. Clin. Gastroenterol..

[89] Haghighat L., Crum-Cianflone N.F. (2016). The potential risks of probiotics among HIV-infected persons: Bacteraemia due to *Lactobacillus acidophilus* and review of the literature. Int. J. STD AIDS.

[90] Sadowska-Krawczenko I., Paprzycka M., Korbal P., Wiatrzyk A., Krysztopa-Grzybowska K., Polak M., Czajka U., Lutyńska A. (2014). *Lactobacillus rhamnosus* GG suspected infection in a newborn with intrauterine growth restriction. Benef. Microbes.

[91] Liu X., Zhao H., Wong A. (2024). Accounting for the health risk of probiotics. Heliyon.

[92] Pruccoli G., Silvestro E., Pace Napoleone C., Aidala E., Garazzino S., Scolfaro C. (2019). Are probiotics safe? *Bifidobacterium* bacteremia in a child with severe heart failure. Infez. Med..

[93] Acuna-Gonzalez A., Kujawska M., Youssif M., Atkinson T., Grundy S., Hutchison A., Tremlett C., Clarke P., Hall L.J. (2023). *Bifidobacterium* bacteraemia is rare with routine probiotics use in preterm infants: A further case report with literature review. Anaerobe.

[94] Sakurai Y., Watanabe T., Miura Y., Uchida T., Suda N., Yoshida M., Nawa T. (2022). Clinical and Bacteriologic Characteristics of Six Cases of *Bifidobacterium breve* Bacteremia Due to Probiotic Administration in the Neonatal Intensive Care Unit. Pediatr. Infect. Dis. J..

[95] McFarland L.V. (2010). Systematic review and meta-analysis of *Saccharomyces boulardii* in adult patients. World J. Gastroenterol..

[96] Poncelet A., Ruelle L., Konopnicki D., Miendje Deyi V.Y., Dauby N. (2021). *Saccharomyces cerevisiae* fungemia: Risk factors, outcome and links with S. boulardii-containing probiotic administration. Infect. Dis. Now..

[97] Hoppu U., Puputti S., Sandell M. (2021). Factors related to sensory properties and consumer acceptance of vegetables. Crit. Rev. Food Sci. Nutr..

[98] Thomas J.J., Holsen L., Van De Water A.L., Becker K.R., Breithaupt L., Burton-Murray H., Asanza E., Gydus J., Palmer L.P., Stern C.M. (2025). Neural response to food cues in avoidant/restrictive food intake disorder. JAMA Netw. Open.

[99] Viswanathan K., Muthusamy S. (2022). Review on the current trends and future perspectives of postbiotics for developing healthier foods. EFood.

[100] Sukumar M. (2025). Harnessing probiotic fermentation to enhance the bioavailability and health impact of dietary phytochemicals. Food Wellness.

[101] Zhang L., Mi S., Liu R., Sang Y., Wang X. (2020). Evaluation of volatile compounds in milks fermented using traditional starter cultures and probiotics based on odor activity value and chemometric techniques. Molecules.

[102] Nissen L., Casciano F., Gianotti A. (2021). Volatilome changes during probiotic fermentation of combined soy and rice drinks. Food Funct..

[103] Bilgiç İ.G., Seyrekoğlu F. (2025). The use of stevia (*Stevia rebaudiana*) as a sweetener in fruit yogurts produced with apple powder and the determination of quality parameters. J. Food Meas. Charact..

[104] Gupta S., Abu-Ghannam N. (2012). Probiotic fermentation of plant-based products: Possibilities and opportunities. Crit. Rev. Food Sci. Nutr..

[105] Tomar O. (2019). The effects of probiotic cultures on the organic acid content, texture profile and sensory attributes of Tulum cheese. Int. J. Dairy Technol..

[106] Guimarães J.T., Balthazar C.F., Silva R., Rocha R.S., Graça J.S., Esmerino E.A., Silva M.C., Sant’Ana A.S., Duarte M.C., Freitas M.Q. (2020). Impact of probiotics and prebiotics on food texture. Curr. Opin. Food Sci..

[107] Han X., Yang Z., Jing X., Yu P., Zhang Y., Yi H., Zhang L. (2016). Improvement of the texture of yogurt by use of exopolysaccharide-producing lactic acid bacteria. BioMed Res. Int..

[108] Solanki H.K., Pawar D.D., Shah D.A., Prajapati V.D., Jani G.K., Mulla A.M., Thakar P.M. (2013). Development of microencapsulation delivery system for long-term preservation of probiotics as biotherapeutic agents. BioMed Res. Int..

[109] Oberoi K., Tolun A., Sharma K., Sharma S. (2019). Microencapsulation: An overview for the survival of probiotic bacteria. J. Microbiol. Biotechnol. Food Sci..

[110] Gallardo-Escamilla F.J., Kelly A.L., Delahunty C.M. (2007). Mouthfeel and flavour of fermented whey with added hydrocolloids. Int. Dairy J..

[111] Godoi F.C., Ningtyas D.W., Geoffroy Z., Prakash S. (2021). Protein-based hydrocolloids: Effect on the particle size distribution, tribo-rheological behaviour and mouthfeel characteristics of low-fat chocolate flavoured milk. Food Hydrocoll..

[112] Vlădescu S.C., Agurto M.G., Myant C., Boehm M.W., Baier S.K., Yakubov G.E., Carpenter G., Reddyhoff T. (2023). Protein-induced delubrication: How plant-based and dairy proteins affect mouthfeel. Food Hydrocoll..

[113] Wang X., Qi Y., Zheng H. (2022). Dietary Polyphenol, Gut Microbiota, and Health Benefits. Antioxidants.

[114] Petersen K., Mansell T.J. (2025). Unveiling the prebiotic potential of polyphenols in gut health and metabolism. Curr. Opin. Biotechnol..

[115] Edgar S., Hopley B., Genovese L., Sibilla S., Laight D., Shute J. (2018). Effects of collagen-derived bioactive peptides and natural antioxidant compounds on proliferation and matrix protein synthesis by cultured normal human dermal fibroblasts. Sci. Rep..

[116] Frøst M.B., Janhøj T. (2007). Understanding creaminess. Int. Dairy J..

[117] Wang Q.J., Mielby L.A., Junge J.Y., Bertelsen A.S., Kidmose U., Spence C., Byrne D.V. (2019). The role of intrinsic and extrinsic sensory factors in sweetness perception of food and beverages: A review. Foods.

[118] Vasudha S., Mishra H.N. (2013). Non-dairy probiotic beverages. Int. Food Res. J..

[119] Zheng X., Yu Y., Xiao G., Xu Y., Wu J., Tang D., Zhang Y. (2014). Comparing product stability of probiotic beverages using litchi juice treated by high hydrostatic pressure and heat as substrates. Innov. Food Sci. Emerg. Technol..

[120] Palencia-Argel M., Rodríguez-Villamil H., Bernal-Castro C., Díaz-Moreno C., Fuenmayor C.A. (2024). Probiotics in anthocyanin-rich fruit beverages: Research and development for novel synbiotic products. Crit. Rev. Food Sci. Nutr..

[121] Lumby N., Park J.J. (2021). Packaging for probiotic beverages. Probiotic Beverages.

[122] Francis D.V., Dahiya D., Gokhale T., Nigam P.S. (2024). Sustainable packaging materials for fermented probiotic dairy or non-dairy food and beverage products: Challenges and innovations. AIMS Microbiol..

[123] Xu R., Tang L., Gao X., Han X., Liu C., Song H. (2025). Study of Aroma Characteristics and Establishment of Flavor Molecular Labels in Fermented Milks from Different Fermentation Strains. Foods.

[124] Tavakoli M., Habibi Najafi M.B., Mohebbi M. (2019). Effect of the milk fat content and starter culture selection on proteolysis and antioxidant activity of probiotic yogurt. Heliyon.

[125] Madilo F.K., Letsyo E., Kortei N.K., Adzinyo O.A., Kunadu A.P. (2025). Production and Quality Characteristics of Starter Culture Fermented Aliha, a Maize-Based Indigenous Fermented Beverage. Int. J. Food Sci..

[126] Gao Y., Liu Y., Ma T., Liang Q., Sun J., Wu X., Song Y., Nie H., Huang J., Mu G. (2025). Fermented Dairy Products as Precision Modulators of Gut Microbiota and Host Health: Mechanistic Insights, Clinical Evidence, and Future Directions. Foods.

[127] Xie A., Dong Y., Liu Z., Li Z., Shao J., Li M., Yue X. (2023). A Review of Plant-Based Drinks Addressing Nutrients, Flavor, and Processing Technologies. Foods.

[128] Tao A., Zhang H., Duan J., Xiao Y., Liu Y., Li J., Huang J., Zhong T., Yu X. (2022). Mechanism and application of fermentation to remove beany flavor from plant-based meat analogs: A mini review. Front. Microbiol..

[129] Yang X., Hong J., Wang L., Cai C., Mo H., Wang J., Fang X., Liao Z. (2024). Effect of Lactic Acid Bacteria Fermentation on Plant-Based Products. Fermentation.

[130] Rachtan-Janicka J., Gajewska D., Szajewska H., Włodarek D., Weker H., Wolnicka K., Wiśniewska K., Socha P., Hamulka J. (2025). The Role of Plant-Based Beverages in Nutrition: An Expert Opinion. Nutrients.

[131] Chen A. (2025). Traditional Fermented Foods and Their Physicochemical, Sensory, Flavor, and Microbial Characteristics. Foods.

[132] Yuan Y., Yang Y., Xiao L., Qu L., Zhang X., Wei Y. (2023). Advancing Insights into Probiotics during Vegetable Fermentation. Foods.

[133] Žvirdauskienė R., Jonikė V., Bašinskienė L., Čižeikienė D. (2025). Fruit and Vegetable Juices as Functional Carriers for Probiotic Delivery: Microbiological, Nutritional, and Sensory Perspectives. Microorganisms.

[134] Rahman M.S., Emon D.D., Toma M.A., Nupur A.H., Karmoker P., Iqbal A., Aziz M.G., Alim M.A. (2023). Recent advances in probiotication of fruit and vegetable juices. J. Adv. Vet. Anim. Res..

[135] Grujović M.Ž., Semedo-Lemsaddek T., Marković K.G. (2025). Application of Probiotics in Foods: A Comprehensive Review of Benefits, Challenges, and Future Perspectives. Foods.

[136] Ren W., Liang H., Li B., Li J. (2025). Towards ideal plant-based yogurts: Evaluating component and processing effects on mouthfeel and stability. J. Future Foods.

[137] Shin J.S., Kim B.H., Kim H.S., Baik M.Y. (2022). Optimization of pea protein and citrus fiber contents for plant based stirred soymilk yogurt using response surface methodology. Food Sci. Biotechnol..

[138] Dhakal D., Kumar G., Devkota L., Subedi D., Dhital S. (2024). The choice of probiotics affects the rheological, structural, and sensory attributes of lupin-oat-based yoghurt. Food Hydrocoll..

[139] Shu N., Chen X., Sun X., Cao X., Liu Y., Xu Y.J. (2023). Metabolomics identify landscape of food sensory properties. Crit. Rev. Food Sci. Nutr..

[140] Chung H.J., Sim J.H., Min T.S., Choi H.K. (2018). Metabolomics and lipidomics approaches in the science of probiotics: A review. J. Med. Food.

[141] O’Connell T.M. (2020). The application of metabolomics to probiotic and prebiotic interventions in human clinical studies. Metabolites.

[142] Kwoji I.D., Aiyegoro O.A., Okpeku M., Adeleke M.A. (2023). ‘Multi-omics’ data integration: Applications in probiotics studies. npj Sci. Food.

[143] Wen L., Yang L., Chen C., Li J., Fu J., Liu G., Kan Q., Ho C.T., Huang Q., Lan Y. (2024). Applications of multi-omics techniques to unravel the fermentation process and the flavor formation mechanism in fermented foods. Crit. Rev. Food Sci. Nutr..

[144] Dong X., Du R., Zhao X., Fang J., Li C., Liu L., Guo W. (2025). Integrated flavoromics and multivariate analysis reveal mechanisms of dynamic changes in characteristic flavors during yogurt fermentation and acidification process. J. Future Foods.

[145] Yu G., Zhang C., Li X., Kong X., Chen Y., Hua Y. (2025). GC×GC-TOFMS and LC-ESI-MS/MS based flavoromics and metabolomics studies on the lactic acid bacteria fermentation of soymilks with dairy milk as the reference. Food Biosci..

[146] Li B., Ye L., Zhao Y., Liu Y., Chen Y., Zhang H. (2025). A comprehensive review of probiotic yogurt: Nutritional modulation, flavor improvement, health benefits, and advances in processing techniques. Agric. Prod. Process. Storage.

[147] Salaün F., Mietton B., Gaucheron F. (2005). Buffering capacity of dairy products. Int. Dairy J..

[148] Kim M., Oh S., Imm J.Y. (2018). Buffering Capacity of Dairy Powders and Their Effect on Yoghurt Quality. Korean J. Food Sci. Animal. Res..

[149] Peri S., Sørensen K.I., da Fonseca C.S., Jensen P.E. (2025). Unlocking the Potential of Oat Okara: A Review of Origins, Composition and Up-Cycling Opportunities in Plant-Based Foods. Future Foods.

[150] Fatima S.M., Hekmat S. (2020). Microbial and Sensory Analysis of Soy and Cow Milk-Based Yogurt as a Probiotic Matrix for *Lactobacillus rhamnosus* GR-1. Fermentation.

[151] Okur H.H., Yıldırım H.K., Yousefvand A., Saris P.E. (2025). Production of Fermented Soy and Soy/Cow Milk Products with Probiotic *Lacticaseibacillus rhamnosus* GG Strain. Food Bioprocess Technol..

[152] Champagne C.P., Raymond Y., Guertin N., Bélanger G. (2015). Effects of storage conditions, microencapsulation and inclusion in chocolate particles on the stability of probiotic bacteria in ice cream. Int. Dairy J..

[153] Sridhar K., Bouhallab S., Croguennec T., Renard D., Lechevalier V. (2023). Recent trends in design of healthier plant-based alternatives: Nutritional profile, gastrointestinal digestion, and consumer perception. Crit. Rev. Food Sci. Nutr..

[154] D’Almeida A.P., Neta A.A.I., de Andrade-Lima M., de Albuquerque T.L. (2024). Plant-based probiotic foods: Current state and future trends. Food Sci. Biotechnol..

[155] Sionek B., Szydłowska A. (2025). Probiotics and prebiotics in the aspect of health benefits and the development of novel plant-based functional food. Appl. Sci..

[156] Shori A.B. (2017). Microencapsulation improved probiotics survival during gastric transit. HAYATI J. Biosci..

[157] Sbehat M., Mauriello G., Altamimi M. (2022). Microencapsulation of probiotics for food functionalization: An update on literature reviews. Microorganisms.

[158] Wang X., Xie W., Zhang S., Shao Y., Cai J., Cai L., Wang X., Shan Z., Zhou H., Li J. (2022). Effect of Microencapsulation Techniques on the Stress Resistance and Biological Activity of Bovine Lactoferricin-Lactoferrampin-Encoding *Lactobacillus reuteri*. Foods.

[159] D’Amico V., Cavaliere M., Ivone M., Lacassia C., Celano G., Vacca M., la Forgia F.M., Fontana S., De Angelis M., Denora N. (2025). Microencapsulation of Probiotics for Enhanced Stability and Health Benefits in Dairy Functional Foods: A Focus on Pasta Filata Cheese. Pharmaceutics.

[160] Holkem A.T., Favaro-Trindade C.S. (2020). Potential of solid lipid microparticles covered by the protein–polysaccharide complex for protection of probiotics and proanthocyanidin-rich cinnamon extract. Food Res. Int..

[161] Yao M., Xie J., Du H., McClements D.J., Xiao H., Li L. (2020). Progress in microencapsulation of probiotics: A review. Compr. Rev. Food Sci. Food Saf..

[162] Stanton C., Ross R.P., Fitzgerald G.F., Van Sinderen D. (2005). Fermented functional foods based on probiotics and their biogenic metabolites. Curr. Opin. Biotechnol..

[163] Costa G.M., Paula M.M., Barão C.E., Klososki S.J., Bonafé E.G., Visentainer J.V., Cruz A.G., Colombo Pimentel T.C. (2019). Yoghurt added with *Lactobacillus casei* and sweetened with natural sweeteners and/or prebiotics: Implications on quality parameters and probiotic survival. Int. Dairy J..

[164] Kimoto-Nira H., Moriya N., Hayakawa S., Kuramasu K., Ohmori H., Yamasaki S., Ogawa M. (2017). Effects of rare sugar D-allulose on acid production and probiotic activities of dairy lactic acid bacteria. J. Dairy Sci..

[165] Ozcan T., Eroglu E. (2022). Effect of stevia and inulin interactions on fermentation profile and short-chain fatty acid production of *Lactobacillus acidophilus* in milk and in vitro systems. Int. J. Dairy Technol..

[166] Pázmándi M., Kovács Z., Maráz A. (2021). Potential of *Lactobacillus* strains for the production of fermented functional beverages enriched in galacto-oligosaccharides. LWT.

[167] Muñoz-Labrador A., Lebrón-Aguilar R., Quintanilla-López J.E., Galindo-Iranzo P., Azcarate S.M., Kolida S., Kachrimanidou V., Garcia-Cañas V., Methven L., Rastall R.A. (2022). Prebiotic potential of a new sweetener based on galactooligosaccharides and modified mogrosides. J. Agric. Food Chem..

[168] Köhler S., Schmacht M., Troubounis A.H., Ludszuweit M., Rettberg N., Senz M. (2021). Tradition as a stepping stone for a microbial defined water kefir fermentation process: Insights in cell growth, bioflavoring, and sensory perception. Front. Microbiol..

[169] Setiarto R.H.B., Octaviana S., Perwitasari U., Juanssilfero A.B., Suprapedi S. (2024). Development of functional bioflavor based on Indonesian indigenous microbial fermentation products. J. Ethn. Foods.

[170] Yangılar F., Cakmakci S. (2017). Probiotic shelf-life, mineral contents and others properties of probiotic yoghurts supplemented with corn flour. J. Agric. Sci..

[171] Kruk M., Lalowski P., Hoffmann M., Trząskowska M., Jaworska D. (2024). Probiotic bacteria survival and shelf life of high fibre plant snack-model study. Plant Foods Hum. Nutr..

[172] Pimentel G., Burton K.J., von Ah U., Bütikofer U., Pralong F.P., Vionnet N., Portmann R., Vergères G. (2018). Metabolic Footprinting of Fermented Milk Consumption in Serum of Healthy Men. J. Nutr..

[173] Thapa D., Kumar V., Naik B., Kumar V., Gupta A.K., Mohanta Y.K., Mishra B., Rustagi S. (2024). Harnessing probiotic foods: Managing cancer through gut health. Food Sci. Biotechnol..

[174] Zolfaghari G., Castro-Alija M.J., Laguillo Diaz M., Ramón-Carreira L.C., Jiménez J.M., Albertos I. (2025). The influence of Mediterranean and Western dietary patterns on sensory perception and taste sensitivity: A study among university students. Foods.

[175] Holck A., Heir E., Johannessen T.C., Axelsson L., Toldrá F. (2014). Northern European products. Handbook of Fermented Meat and Poultry.

[176] García-Barón S.E., Carmona-Escutia R.P., Herrera-López E.J., Leyva-Trinidad D.A., Gschaedler-Mathis A. (2025). Consumers’ drivers of perception and preference of fermented food products and beverages: A systematic review. Foods.

[177] Roose G., Mulier L. (2020). Healthy advertising coming to its senses: The effectiveness of sensory appeals in healthy food advertising. Foods.

[178] Tonacci A., Billeci L., Di Mambro I., Marangoni R., Sanmartin C., Venturi F. (2021). Wearable sensors for assessing the role of olfactory training on the autonomic response to olfactory stimulation. Sensors.

[179] Billeci L., Sanmartin C., Tonacci A., Taglieri I., Bachi L., Ferroni G., Braceschi G.P., Odello L., Venturi F. (2023). Wearable sensors to evaluate autonomic response to olfactory stimulation: The influence of short, intensive sensory training. Biosensors.

[180] Spence C., Chen J., Engelen L. (2012). Multi-sensory integration and the psychophysics of flavour perception. Food Oral Processing: Fundamentals of Eating and Sensory Perception.

[181] Billeci L., Sanmartin C., Tonacci A., Taglieri I., Ferroni G., Marangoni R., Venturi F. (2025). Wearable sensors to measure the influence of sonic seasoning on wine consumers in a live context: A preliminary proof-of-concept study. J. Sci. Food Agric..

[182] Bayarri S., Calvo C., Costell E., Durán L. (2001). Influence of color on perception of sweetness and fruit flavor of fruit drinks. Food Sci. Technol. Int..

[183] Wang Q.J., Spence C. (2018). Sonic packaging: How packaging sounds influence multisensory product evaluation. Multisensory Packaging: Designing New Product Experiences.

[184] Liu S., Gu Y., Zheng R., Sun B., Zhang L., Zhang Y. (2024). Progress in multisensory synergistic salt reduction. Foods.

